# Salmagundi

**Published:** 1887-06

**Authors:** 


### SALMAGUNDI.

EDITED BY E. G. BETTY, D.D.S.

THE MAD RIVER DENTAL SOCIETY held no meeting last month, the postponement being made on account of a fusion meeting with the State Society—October next, at Springfield.

McKellop's Dental Blotter.—This registering ledger is just issued and constitutes one of the best offered to the profession. The diagram includes a cut of each tooth, root and all, so that any operation upon any part of the tooth may be recorded. It is a neat book of three hundred pages, with capacity for that many cases; debit and credit columns, names, dates, etc. In addition to this each case is recorded upon one leaf, which is perforated at the left, so that if necessary, it may be torn out and given to the patient or preserved. It is published by R. & T. A. Ennis, Nos. 118 and 120 Olive street, St. Louis, Mo., of whom it may be obtained.

Urethane.—Dr. Andrews has conducted some interesting experiments with this hypnoptic. Having first tested the effects of the remedy upon himself, he proceeded to try it in six cases of acute mania, four of paresis, four of melancholia, two of chronic mania, and two of dementia. The results of these experiments were on the whole decidedly favorable; but the author justly observes that before definite conclusions can be arrived at a more extended series of trials is necessary. Irrespective of this fact, however, the results obtained by Andrews are decidedly encouraging. The dose usually employed by him was thirty grains. On two occasions, however, doses of sixty grains were given. The result of these observations goes to show that urethane possesses marked hypnotic properties. Throughout these experiments no unpleasant effects were observed, and there were no phenomena pointing to other than an action affecting the cerebrum. The effects of the drug were felt, so far as Andrews was able to estimate them, within an hour after its administration.

Conditions of Successful Production of Local Anæsthesia in Tooth Extraction.—Dr. A. Lebrun, of Brussels, and his house-surgeon M. Andries, have published the notes of twenty-nine cases in which they employed local cocaine anæsthesia for the extraction of teeth. In twenty-three cases the anæsthesia was complete, in one case partial. In the remaining five cases the procedure was unsuccessful. This is attributed by them to the difficulty of introducing the short straight needle of the hypodermic syringe in the case of the second and third molars, and also perhaps to no precautions having been taken to prevent the escape of the liquid. They point out that M. Viau, of Paris, who succeeded in producing a complete anæsthesia in every one of his eighty-six cases, made use of a specially constructed syringe, with a holder by which it could be grasped firmly between the index and middle fingers, and provided with needles of various degrees of curvature; also that he surrounded the tooth with plugs of cotton-wool, and applied the finger over the puncture after the needle. Beside which he made the patient keep rinsing his mouth with cold water during the five minutes that intervened betwen the introduction of the cocaine and the actual operation. These precautions were not observed by M M. Lebrun and Andries. They used, however, a solution similar to that of M. Viau—viz., fifty centigrammes (seven and a half minims) of a 2 per cent. solution of carbolic acid containing five centigrammes (three-fourths of a grain) of hydrochlorate of cocaine, half of which quantity was injected into each surface of the gum.—*Lancet*, March 19, 1887.

---

*Items of Interest* has an editorial in the May number upon prompt collections that in the main are good. The only objection to it is this sentence, "Don't be afraid of letting your patient know before you begin how much their work is to cost as near as may be, and when you expect to be through." There are two reasons that experience has shown that proposition to be wrong. First, it encourages "shopping," i. e., people running about from one dentist to another seeking the one who will underbid, and tattling what they hear. Second, that it greatly hampers the dentist in his work, for, as is very frequently the case, unsuspected cavities will be found or some unforseen complication will arise that necessitates a much greater expenditure of time and material than calculated upon, and if a price has been fixed, either the dentist must work at a sacrifice or shirk what is a manifest duty, even though it entails upon him a financial loss. It is far better to "make a friend" of the patient, gain his confidence and with that once obtained, no matter what the work is that must be done or what the cost incurred, the patient will rarely question the amount of the bill. It sometimes happens though, that, even if the price of any one of a number of fillings should not exceed five or six dollars, yet, a large number of them will make the bill seem exhorbitantly large and the patient may be disposed to dispute it, yet when shown the diagram in the registering ledger and the analysis of the bill by items, he then sees that it is merely a question of the amount of work done.

